# Bariatric arterial embolization in patients with body mass index ranging from 25 to 40 kg/m^2^: A systematic review & meta-analysis

**DOI:** 10.34172/jcvtr.2023.32900

**Published:** 2023-12-30

**Authors:** Rishabh Khurana, Niraj Nirmal Pandey, Sanjeev Kumar, Priya Jagia

**Affiliations:** Department of Cardiovascular Radiology & Endovascular Interventions, All India Institute of Medical Sciences, New Delhi, India

**Keywords:** Bariatrics, Weight loss, Body mass index

## Abstract

The present review sought to evaluate by meta-analysis the efficacy of bariatric arterial embolization (BAE) in promoting weight loss in patients with body mass index (BMI) ranging from 25-40 kg/m^2^. This study was performed and reported according to Preferred Reporting Items for Systematic reviews and Meta-analysis guidelines. A systematic literature search of MEDLINE, Embase Google Scholar, and World Health Organization Library database was done for studies evaluating BAE for promoting weight loss in patients with BMI ranging from 25-40 kg/ m^2^ published up to March 10, 2021. Primary outcome measure included weight loss after the embolisation procedure. Three single-arm studies comprising of a total of 28 patients (BMI: 25- 40 kg/m^2^) were found eligible for meta-analysis. All patients underwent embolization with either Embosphere microspheres or PVA particles. The predominant artery embolised was left gastric artery (in all patients). Additional arteries embolised included gastroepiploic artery (8 patients), or accessory left gastric artery (1 patient), or short gastric artery (1 patient). Pooled absolute mean weight loss was 7.854 kg (95% CI: 6.103-9.605). No significant statistical heterogeneity was detected (I^2^=51.75%, *P*=0.126) among pooled studies. In conclusion, limited single-arm studies report BAE as an effective, and relatively safe procedure for promoting weight loss in patients with BMI ranging from 25-40 kg/m^2^, although the number of patients included is very small. Initial results of BAE in promoting weight loss are promising with no major/severe complications reported; however, long term follow-up is required to see the sustainability of the effects.

## Introduction

 Obesity is one of the most widespread public health issues of 21st century and a major cause of morbidity and mortality.^1^ Apart from being just a cosmetic botheration, it is also a medical problem which increases the risk of other ailments including hypertension, diabetes, cardiovascular disease and certain cancers.^2-6^ The therapeutic approaches adopted for weight loss range from dietary alterations, clubbing with regular exercise with or without behaviour interventions, and pharmacotherapy at one end of the spectrum, to bariatric surgery on the other end.^7-14^

 Gastric artery embolization has been part of management of gastrointestinal haemorrhage for a long time and significant weight loss was observed as a side effect in such patients.^15-17^ Subsequently, researchers actively studied the role of gastric artery embolization in promoting weight loss. The underlying hypothesis includes reduction in blood supply to the fundus leading to reduced serum ghrelin levels eventually leading to reduced appetite and promoting weight loss. Moreover, left gastric artery (LGA) embolization indirectly affects production of gastric acid as well as gastric motility and absorption.^18^ In the past decade, various studies have shown bariatric arterial embolization as a promising minimally invasive alternative in the treatment of obese patients, reluctant to undergo bariatric surgery.^18-24^

 Although bariatric surgery is indicated in adult patients with body mass index (BMI) ≥ 40 kg/m^2^ and in adults with BMI ranging from 35.0 to 39.9 kg/m^2^ with at least one serious comorbidity (including but not limited to type II diabetes mellitus, hypertension, obstructive sleep apnea, obesity-hypoventilation syndrome, hyperlipidemia non-alcoholic fatty liver disease, non-alcoholic steatohepatitis, gastroesophageal reflux disease, asthma, debilitating arthritis, impaired quality of life), there is no long-term advantage to justify bariatric surgery in adults with BMI between 30.0 to 34.9 kg/m^2^ with comorbid conditions (type 2 diabetes mellitus, metabolic syndrome).^24-25^ Moreover, bariatric surgical procedures have potential to cause complications.

 Introduction of BAE in overweight and obese patients (BMI: 25 to 40 kg/m^2^) may be a middle path strategy in cases where lifestyle modification has failed and patients are not willing for surgical procedures (due to risk of complications) or pharmacotherapy (due to risk of possible adverse effects). The present systematic review and meta-analysis sought to analyse and demonstrate the feasibility, efficacy and safety of BAE in promoting weight loss, specifically in patients with BMI ranging from 25 to 40 kg/m^2^.

## Material and Methods

 A systematic review and subsequent meta-analysis of several studies assessing the role of BAE in patients with BMI 25 to 40 kg/m^2^, was performed to summarize the patient demographic profile, the embolization techniques including the arteries embolised and the choice of embolising agent, the safety and efficacy of BAE in promoting weight loss.

###  Search strategy

 Ethical approval was not required for performing the systematic review and meta-analysis as per institute policy. The present systematic review and meta-analysis was performed according to Preferred Reporting Items for Systematic reviews and Meta-Analysis (PRISMA) guidelines.^26^ The protocol was registered with the international prospective registry of systematic reviews.

 We performed a comprehensive electronic search of four databases including PubMed, Embase, Google Scholar, and World Health Organization Library database, for studies evaluating BAE for promoting weight loss in patients with BMI ranging from 25-40 kg/m2 published up to March 10, 2021, using the following search terms: “bariatric embolization’, “bariatric artery embolization” and “left gastric artery embolization”. Ancillary search of the grey literature was also performed. Additionally, reference lists of the extracted selected studies were explored, in order to extract other relevant studies. Duplicates were excluded.

###  Study Selection

 The published articles evaluating BAE for promoting weight loss in patients with BMI ranging from 25-40 kg/m^2^ were included in the review. The reports were evaluated using PICO (Population, Intervention, Comparison and Outcomes). For inclusion, the studies had to report weight change (absolute or percentage) in patients with BMI ranging from 25 to 40 kg/m^2^. Additional inclusion criteria were studies conducted on human beings, published in English language and with extractable full text. No restrictions were applied based on the country of research. We excluded case reports, case series with < 5 patients, reviews, editorials, guidelines, preprints, expert opinions and recommendations. Two independent reviewers (RK and NNP) screened the titles and abstracts of the included articles according to the criteria mentioned above. Any disagreement regarding the inclusion of the study in the final analysis was resolved by consensus after discussing with senior author (PJ). A total of 6 studies were selected for full-text evaluation against the pre-determined inclusion and exclusion criteria. Of the 6 eligible studies, 3 studies were found eligible for inclusion in the meta-analysis.

###  Quality of study assessment (risk of bias in individual studies)

 Two independent reviewers (RK and NNP) assessed the methodologic quality of each study independently by National Institutes of Health (NIH) Quality Assessment Tool for Case Series Studies.^27^ Any disagreement was settled by consensus after consulting the senior author (PJ).

###  Data Extraction

 Full texts of the 6 articles included for the final review were retrieved. These were further screened for their eligibility. After careful scrutiny, the articles included for systematic review and subsequent meta-analysis were shortlisted. Extraction of the relevant data from the full texts of the included articles, into a Microsoft Excel database was conducted by two independent reviewers. The fields included: author, year of publication, country, journal, study design, sample size, period of study, duration of follow-up. Subsequently, the study population was analysed to determine the demographic data: mean age, gender, mean baseline weight (kg) and BMI (kg/m^2^), dietary considerations, previous/post procedure imaging, biochemical markers (Ghrelin, Leptin, HbA1c: baseline & follow-up), vascular access, hardware used (sheath, catheter, guidewire, microcatheter), embolisation techniques, artery embolised, embolising agent used and periprocedural complications. Adverse events were graded according to Society of Interventional Radiology (SIR) or Cardiovascular and Interventional Radiological Society of Europe (CIRSE) Classification System.^28^ Any disagreement regarding the inclusion of the study in the final analysis was resolved by consensus after discussing with senior author (PJ).

###  Summary measures, results synthesis & statistical analysis

 The primary outcome of interest was absolute weight loss. The pre- and post-procedural data on weights was used to calculate mean weight change (in kg) along with 95% confidence interval (CI). Random-effects models were utilised to estimate pooled results, because it could not be assumed that all included studies were identical.^29^ Substantial variations existed in patient demographic data and baseline parameters. The true statistical heterogeneity of the existing variation was analysed using I^2^ values. Standard error of the mean was estimated for individual study. Standardized mean differences were estimated and pooled. All analyses were performed using R Statistical Software v4.0.0. *P* values < 0.05 were considered significant.

## Results

###  Literature search and study selection

**Figure 1:**
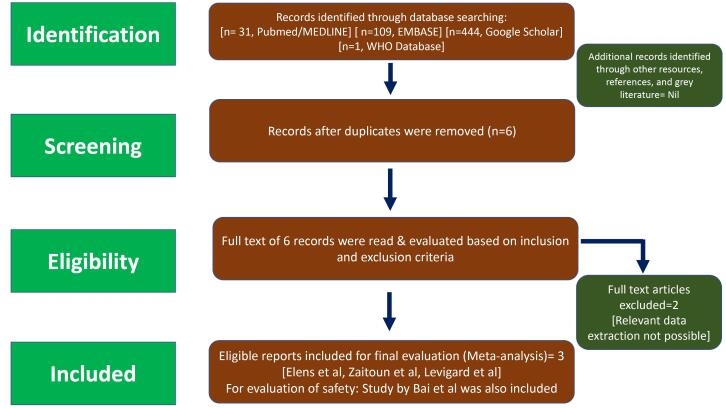
PRISMA (Preferred Reporting Items for Systematic reviews and Meta-Analysis) flow diagram of study selection process

 The study selection process is demonstrated in Figure 1. The included studies were published between October 2017 to January 2021. Four studies were found to satisfy our eligibility criteria. For evaluating the efficacy, three non-randomised prospective trials (single arm), comprising of 28 patients (BMI: 25 to 40 kg/m^2^), were considered for the meta-analysis.^21,30-31^ The various characteristics (author, design, number of patients, indication, follow-up duration along with name of institution where study was conducted) of the included study is depicted in Table 1.

**Table 1:** Study characteristics with baseline characteristics of study population and treatment variables

**Characteristics**	**Elens et al **^21^**, 2019**	**Zaitoun et al **^30^**, 2019**	**Levigard et al **^31^**, 2021**
Country	Belgium	Egypt	Brazil
Study Design	Prospective Single arm February 2015 - May 2017	Prospective Single arm January 2017-June 2018	Prospective Single arm March-August 2018
Sample size of the study	11	10	10
Sample Size qualifying our inclusion criteria	11	7***	10
Indications & BMI Criteria	Obesity (BMI 25-30)	Obese & Prediabetic (BMI > 30) HbA1c: 5.7-6.4	Obese, Non-menopausal woman with metabolic syndrome. BMI: 30-39.9
Follow-up period/month	Up to 6 months	Up to 6 months	Up to 6 months
Age (years)	38 ± 10	37.14 ± 8.44^#^	37.5 ± 7.26
Sex Ratio	87% Female	85.7% Female^#^	All Females
Baseline weight (kg)	79 ± 10	102.89 ± 11.83^#^	94.30 ± 7.21
Baseline BMI (kg/m^2^)	28.9 ± 2.5	35.73 ± 2.48^#^	36.37 ± 2.58
Dietary Consideration	No	No	9 out of 10 patients didn’t follow the proposed low-calorie dietary plan after first month following the procedure.
Biochemical parameters	Not evaluated	Mean HbA1c: 5.9 ± 0.14	Acylated ghrelin levels Homeostatic Model Assessment- Insulin Resistance (HOMA-IR)
Other parameters (Including Patient Reported Scores/Scale)	Hunger sensation and satisfactory scale reviewed	Not evaluated	Waist Circumference Binge Eating Scale (BES) Word Health Organization Quality of Life (QOL) questionnaire, (WHOQOL-BREF)

***Although study’s total sample size was 10, however, 3 patients (2 males & 1 female) were excluded as their BMI were > 40 kg/m^2^. Hence, effective sample size was 7 for the purpose of the evaluation. ^#^The study characteristics were re-calculated.

###  Quality assessment of the studies

 The results of National Institutes of Health (NIH) Quality Assessment Tool for Case Series Studies are presented inTable 2. The quality rating of all included studies was good.

**Table 2:** Evaluating Risk of Bias of Included Studies Using National Institutes of Health (NIH) Quality Assessment Tool for Case Series Studies

	**Criteria**	**Elens et al **^21^	**Zaitoun et al **^30^	**Levigard et al **^31^
1.	Was the study question or objective clearly stated?	Yes	Yes	Yes
2.	Was the study population clearly and fully described, including a case definition?	Yes	Yes	Yes
3.	Were the cases consecutive?	Yes	Yes	Yes
4.	Were the subjects comparable?	Yes	Yes	Yes
5.	Was the intervention clearly described?	Yes	Yes	Yes
6.	Were the outcome measures clearly defined, valid, reliable, and implemented consistently across all study participants?	Yes	Yes	Yes
7.	Was the length of follow-up adequate?	No	No	No
8.	Were the statistical methods well-described?	Yes	Yes	Yes
9.	Were the results well-described?	Yes	Yes	Yes
Response: Yes/No/ Other (CD, cannot determine; NA, not applicable; NR, not reported)				
Quality Rating (Good/Fair/Poor) Rater #1 initials: RK Rater #2 initials: NNP Additional Comments (If POOR, please state why)		RK: Good NNP: Good	RK: Good NNP: Good	RK: Good NNP: Good

###  Patient characteristics

 All included patients were adults, with female preponderance (84.9%). The baseline characteristics of study population and therapeutic variables are depicted in Table 1.

###  Embolization procedure

 The most common access used was retrograde femoral using a 5F sheath. Left distal radial artery access was used in 8 patients.^31^ For selective angiography of celiac or superior mesenteric artery, a cobra catheter was used. The catheters used subsequent to radial access included Judkins Right Coronary 4 (Terumo, Tokyo, Japan), Internal Mammary (Terumo, Tokyo, Japan) and Cobra 2 catheter (Cordis, FL, USA).^31^ All arteries supplying the gastric fundus including the LGA and other potential accessory gastric arteries were identified.

 The various microcatheters used included Progreat (2.7/2.8F; Terumo Corp, Tokyo, Japan); Excelsior 1018 Microcatheter (Boston Scientific Corp, Cork, Ireland) and Renegade HI-FLO microcatheter (Boston Scientific, Marlborough, Massachusetts). In two patients, retrograde catheterisation of LGA was performed via superior mesenteric artery and gastroduodenal artery to reach the LGA (due to early origin of LGA on the celiac trunk). Diagnostic angiogram of LGA was performed to look for fundal perfusion defects to indicate a collateral supply to fundus and need for embolisation of the corresponding artery as well.

 The technical aspects of the procedure (including artery embolised, embolising agent used, particle size, end point) have been summarised in Table 3.

**Table 3:** Technical details of bariatric arterial embolization procedure across included studies

**Technical aspects**	**Elens et al **^21^** (n=11)**	**Zaitoun et al **^30^ (n=7/10)**	**Levigard et al **^31^** (n=10)**
Access	Femoral Artery	Femoral Artery	Right Femoral artery (2 patients) Left distal radial artery (8 patients)
Embolising Agent	Embosphere microspheres	Embosphere microspheres	Embosphere microspheres
Particle Size	500–700 μm (300–500 μm in 2 cases only)*	300–500 μm	300–500 μm
End Point	Embosphere injection was alternated with contrast (until distal branches of LGA were no longer visible). This was followed by Gelfoam to prevent reflux.	Stasis	Stasis
Artery Embolised	Left Gastric Artery	Left Gastric Artery	Left Gastric Artery (all patients) Additional Branches: Gastroepiploic artery (n = 8); accessory left gastric artery (n = 1); short gastric artery (n = 1)

*These were not used subsequently in this study due to severe pain and safety concerns **Although study’s total sample size was 10, however, 3 patients (2 males & 1 female) were excluded as their BMI were > 40 kg/m^2^. Hence, effective sample size was 7 for the purpose of the evaluation.

###  Clinical outcome

**Figure 2:**
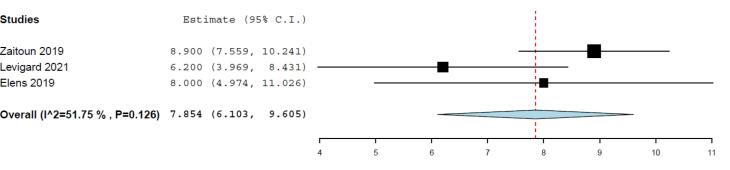
Random effects forest plot of weight loss across studies

 Pooled absolute mean weight loss was 7.854 kg (95% CI: 6.103 to 9.605) ranging from 6.2 kg **to** 8.9 kg. At-least 6 months follow-up was present. No significant statistical heterogeneity was detected (I^2^ = 51.75%, *P* = 0.126) among pooled studies (Figure 2, Table 4).

**Table 4:** Pooled analysis of weight loss after bariatric arterial embolization procedure

**Author**	**Absolute Weight Loss**		
	**Mean weight loss (kg)**	**95% CI Lower Limit**	**95% CI Upper Limit**
Elens et al ^21^	8	4.97	11.03
Zaitoun et al ^30^	8.9	7.559	10.24
Levigard et al ^31^	6.22	4.97	11.02
Pooled weight loss	7.854	6.103	9.605

###  Adverse events

 Different criteria were used to categorize the complications. The criteria along with various complications have been depicted in Table 5. The most common adverse events were superficial gastric ulceration and mild epigastric pain. Pooled analysis of various adverse events could not be conducted due to non-availability of patient-specific data.

**Table 5:** Various complications observed across included studies

**Complication**	**Elens et al **^21^** (n=11)**	**Zaitoun et al **^30^ (n=7/10)*	**Levigard et al **^31^** (n=10)**
Criteria used		Society of Interventional Radiology	Updated Clavien-Dindo classification for surgical complications
Major complication	1/11^$^	Not reported	Not reported
Minor Complications			
Superficial gastric ulceration	1/11	No event detected	8
Deep gastric ulceration	No event detected	No event detected	2
Mild epigastric pain	Not reported	7 ** 7 out of 10 patients	Not reported
Puncture site related complication	Not reported	Not reported	1

*Total Sample size was 10. However, only 7 patients qualified our inclusion criteria. ** Exact patient wise details were not provided. $: Definition of major complication: adverse events leading to either admission in ICU or a longer hospital stay

 Superficial gastric ulceration was present in 1 out of 11 patients (Elens et al), and 8 out of 10 patients (Levigard et al). Other minor complications included mild epigastric pain and puncture site related complication. Major adverse event (at least grade 3 according to CIRSE classification) was reported in 1 of the patients which included severe pancreatitis, splenic infarction and late gastric perforation which necessitated hospitalisation for 1 month (Elens et al). No major adverse event was detected in rest of the included studies.

## Discussion

 According to the World Health Organization, there are approximately 650 million obese adults worldwide. According to BMI, patients are categorised as overweight (BMI ranging from 25 to 29.9 kg/m^2^), obese (BMI ranging from 30 to 34.9 kg/m^2^), and severely obese (BMI > 40 or > 35 along with obesity-related comorbidity).^1^ The first line of management for overweight or obese patient is lifestyle interventions which include dietary modification, modifying eating behaviour, and physical activity). This conservative approach can achieve an average weight reduction of 5–10% in overweight and obese patients (BMI between 25-29.9 kg/m^2^, 30-34.9 kg/m^2^, respectively).^6,7^ However, various studies have depicted that these individuals may regain weight in a small span of time after initiating conservative strategies. Given the fact that lifestyle changes and pharmacological therapy are often ineffective, more and more patients are opting for bariatric surgery. Bariatric surgeries are indicated in severely obese/morbidly obese patients.^8-14^

 To mitigate the various complications attributed to bariatric surgery, several studies across the globe have been conducted in the past decade demonstrating the feasibility, safety and efficacy of a novel minimally invasive technique i.e., bariatric arterial embolization.^18-23,30-31^ Having observed encouraging results (in terms of significant weight loss), several issues remain unclear. These include ideal patient selection, existing comorbidities, standardising the pre-procedural investigations, role of pre-procedural imaging, biochemical markers, hardware selection, angiography technique, vessel to target, ideal embolisation agent used (and particle size), end point of embolisation, peri-procedural complications, and access site haemostasis.

###  Study and patient characteristics

 Three non-randomised clinical trials were included which consisted of patients fulfilling the inclusion criteria of our systematic review. These comprised of a total of 28 patients (BMI 25 to 40 kg/m^2^), who underwent BAE for weight loss. Female preponderance was seen. Follow-up data for 6 months was available. The pooled mean weight loss at 6 months was 7.854 kg. Previous systematic review and meta-analysis had shown an average weight loss of 8.85 kg ± 1.24 kg in 12 months, in a population with mean BMI of 41.05 kg/m^2^, and men were more likely than women to lose weight after the procedure.^32^ Although, all the bariatric trials included in our study comprised of subjects who were overweight or obese (BMI 25-40 kg/m^2^), substantial variations were present in study design and population characteristics.

 Most of the earlier studies evaluating this novel approach have targeted patients with severe obesity. Elens et al were the first to evaluate BAE in overweight patients (not candidates for bariatric surgery) and reported a mean weight reduction of 10% at 3 & 6 months. Zaitoun et al evaluated obese and prediabetic individuals. Statistically significant weight loss was observed in 6 months with a mean percent reduction in body weight of 8.9%. Additionally, there was a significant fall in HbA1c levels (mean reduction: 21.4%) leading to resolution of prediabetic state in all patients (although long term data and sustainability of this effect is not available yet). A previously published meta-analysis had stated that the only variable positively associated with weight loss after bariatric artery embolisation procedure, was the male gender. Subsequently, Levigard et al evaluated the safety and efficacy of this therapeutic approach in a female cohort with class I and II obesity along with metabolic syndrome. The observed mean weight loss was 6.2 kg (slightly lower than above-mentioned two studies) despite a more extensive embolisation approach (targeting more than one vessel). Failure to follow dietary instructions, lack of psychological counselling, long work hours and economic issues were the various probable reasons quoted to explain the above effect. Other parameters assessed were serum ghrelin levels, waist circumference, insulin resistance, binge eating scale (BES), and quality of life (QOL) questionnaire. Drop in ghrelin levels was approximately 49% higher than previously published results. Moreover, there was a remarkable impact on glucose metabolism (normalisation of fasting glucose and reduction in insulin resistance), with a persistent improvement in QOL and BES.

 All the included studies had excluded patients with history of peptic ulcer disease (or endoscopy revealing active ulcer), prior gastric surgery, renal or hepatic dysfunction.

###  Angiography procedure and embolization technique

 The most common access site used was femoral using 5F sheath, followed by left radial approach. The feasibility of LGA angiography via transradial approach has been shown in cohort of coronary artery disease patients having obesity.^23^ No safety issues were encountered. Levigard et al reported left distal radial artery thrombosis in 1 patient; which was without any clinical sequalae.

 Elens et al had used 500-700 μm Embosphere microspheres (Merit medical, Jordan, Utah) particles after observing severe pain reported by initial patients [embolized with smaller sized particles (300–500 μm) particles]. The probable reason of pain suggested was smaller size of particles leading to more distal penetration as well as splenic infarcts. However, in subsequent studies by Zaitoun et al and Levigard et al 300–500 μm embosphere microsphere particles (Merit medical, Jordan, Utah) were utilised for embolisation purpose, as by the time of these trials were conducted, 300-500 μm spheres was the smallest particle size deemed to be safe as an embolising agent.

###  Adverse events

 According to the Society of Interventional Radiology, major complications include adverse events requiring hospitalization or resulting in permanent sequelae or death.^33^ Only 1 patient, had a major complication (at least grade 3 according to latest CIRSE classification), presenting as severe pancreatitis, splenic infarction, and gastric perforation requiring intensive care unit stay as well as a prolonged hospital course.^21^ Other complications were categorised into minor (Grade 1 or 2 according to latest CIRSE classification) as described previously.^28^ The extensive embolisation carried out by Levigard et al may explain the increased incidence of superficial gastric ulceration in their study. However, most ulcers, including deep ones, occurred in the transition from the lesser curvature into the gastric fundus. No ulceration was seen in the fundic region.

 The present study had the following limitations. Firstly, only a small number of studies were included in the review. Secondly, there was heterogeneity in the patient inclusion criteria, treatment technique (artery embolised, size of embolising agent), and reported outcomes. Long-term follow-up was lacking. Moreover, a lack of patient specific data in all the studies does not allow for a comprehensive evaluation. Thirdly, pooled analysis of various adverse events could not be performed due to non-availability of patient specific data. Included studies have used different biochemical parameters and various assessment scales which also preclude comparison. Lastly, double blinded randomised controlled trials are lacking.

## Conclusion

 BAE is an effective, and relatively safe procedure for promoting weight loss in overweight and obese (Class I and II) individuals. Although the results are promising in the present studies, and there are no major/severe complications, long term follow-up is required to see the sustainability of the effects. Adopting BAE as a middle path regimen, in reducing weight in overweight and obese individuals who have failed lifestyle modifications and do not fit the inclusion criteria of undergoing bariatric surgeries, seems a plausible option and merits investigation with large-scale, multicentric, double-blinded randomized controlled trials with a long follow-up period.

## Acknowledgements

 None.

## Competing Interests

 The author(s) declared no potential conflicts of interest with respect to the research, authorship, and/or publication of this article.

## Ethical Approval

 Not applicable.

## Funding

 Authors received no financial support for the research, authorship, and/or publication of this article.
